# The Human Factor: Behavioral and Neural Correlates of Humanized Perception in Moral Decision Making

**DOI:** 10.1371/journal.pone.0047698

**Published:** 2012-10-17

**Authors:** Jasminka Majdandžić, Herbert Bauer, Christian Windischberger, Ewald Moser, Elisabeth Engl, Claus Lamm

**Affiliations:** 1 Social, Cognitive and Affective Neuroscience Unit, Faculty of Psychology, University of Vienna, Vienna, Austria; 2 Center for Medical Physics and Biomedical Engineering, Medical University of Vienna, Vienna, Austria; University of Bologna, Italy

## Abstract

The extent to which people regard others as full-blown individuals with mental states (“humanization”) seems crucial for their prosocial motivation towards them. Previous research has shown that decisions about moral dilemmas in which one person can be sacrificed to save multiple others do not consistently follow utilitarian principles. We hypothesized that this behavior can be explained by the potential victim’s perceived humanness and an ensuing increase in vicarious emotions and emotional conflict during decision making. Using fMRI, we assessed neural activity underlying moral decisions that affected fictitious persons that had or had not been experimentally humanized. In implicit priming trials, participants either engaged in mentalizing about these persons (Humanized condition) or not (Neutral condition). In subsequent moral dilemmas, participants had to decide about sacrificing these persons’ lives in order to save the lives of numerous others. Humanized persons were sacrificed less often, and the activation pattern during decisions about them indicated increased negative affect, emotional conflict, vicarious emotions, and behavioral control (pgACC/mOFC, anterior insula/IFG, aMCC and precuneus/PCC). Besides, we found enhanced effective connectivity between aMCC and anterior insula, which suggests increased emotion regulation during decisions affecting humanized victims. These findings highlight the importance of others’ perceived humanness for prosocial behavior - with aversive affect and other-related concern when imagining harming more “human-like” persons acting against purely utilitarian decisions.

## Introduction

Humans are deeply social animals - with highly developed capacities for living in complex groups, such as reciprocity [1] and coalition formation to compete with other individuals or groups [2], [3]. This has greatly advanced and shaped their ability to consider the beliefs, thoughts and feelings of conspecifics, referred to as mental states. “Mentalizing” about others seems a pivotal human skill [4] and even extends to the tendency to attribute an internal mental life to objects that do not have intentions or emotions, such as computers or moving dots, termed anthropomorphism [5]. At the other extreme, people might deliberately deny internal mental states to other people, representing them as objects or animals. Such denial of typically or uniquely human characteristics to others is referred to as dehumanization [6].

The extent to which a person is regarded as a full-blown individual with mental states, i.e., his or her “perceived humanness”, is a central concept in social psychology. People seem to systematically “humanize” their ingroups, attributing more uniquely human characteristics such as complex emotions to their own group than to other groups [7], while outgroup members might be dehumanized, as in historic descriptions of ethnic others as barbarians [8]. The perceived humanness of others also relates to the degree to which they are viewed as deserving moral consideration, and seems to play a role in justifying (as in war situations) or rejecting (as in groups with high social cohesion) harmful acts towards others [9].

This relationship between perceived humanness and moral care might be explained by the notion that people engage in simulation processes to represent and understand other people’s affective states. According to the “shared representations account” of empathy, “feeling with” other people recruits the same neural structures as those involved in the first-hand experience of these affective states [10], [11]. Thus, a stronger a priori perception of the other as a human being with mental states, i.e., as a person whose behavior easily “maps” onto one’s own mind, might evoke a more intense embodied representation of the consequences of a harmful act to this person, resulting in a reluctance to engage in this harmful act.

The effect of others’ perceived humanness on the tendency for prosocial behavior towards them might also be at work during decision making about hypothetical situations in which one person’s life would have to be sacrificed to save the lives of numerous others. In such so-called moral dilemmas, two moral values (in this case “do not kill” vs. “save as many lives as possible”) strongly conflict, and no formal moral principle exists that a priori establishes the appropriate decision or action [12]. In these types of dilemmas judgments can either conform to utilitarian principles, i.e., maximizing the overall “good” outcome of the action, or non-utilitarian values, i.e., refraining from sacrificing one individual (and hence accepting the death of numerous others). Moral dilemma paradigms have been widely used to assess regularities in people’s moral judgments and to identify the principles underlying them. Importantly, it has been shown that choices for either the utilitarian or the non-utilitarian decision option strongly depend on the characteristics of the dilemma situation, implying that the outcome of the decision is not the only factor determining respondents’ choices.

This phenomenon is illustrated by decision patterns in the trolley dilemma [13], [14]. In the “switch” version of this dilemma, one has to imagine seeing a trolley that is running out of control down a track, and is about to run over five rail workers. If a switch is turned, the trolley will be led down a different track and only run over a single rail worker there. When confronted with this dilemma, most people indicate that the utilitarian response option to pull the switch is more appropriate in this situation. In a modification of the dilemma called the “footbridge” dilemma the same outcome (i.e., saving the five persons by sacrificing a single person) can be accomplished by pushing a man with a heavy rucksack off a footbridge onto the track. Here the majority of people prefer the non-utilitarian alternative by indicating that it is not acceptable to push the man off the bridge to save the lives of the five others.

Various suggestions have been made to explain the findings that the utilitarian decision option is judged acceptable in some cases and not in other cases. Suggested factors have been whether the action requires direct physical contact with the victim [15], the spatial proximity to the victim [16], whether the victim is harmed by a mechanic device or by direct physical contact [17], or an interaction between some of these factors [16]. In addition, Greene and colleagues have distinguished between “impersonal” and “personal” moral violations, where the latter refers to an “agent-authored” action that causes serious bodily harm to a particular person and is therefore judged as less morally acceptable [18], [19].

What many of these explanations bear in common is the extent to which the decision forces one to conceive of the victim as a human being when imagining to perform the utilitarian, yet harmful act. In the case of the footbridge dilemma, imagining to push the man off the bridge will automatically elicit a representation of one’s interaction with his body, an image of his face, and thereby the expressions of his affective states (such as shock or fear) to a far greater extent than in the switch dilemma. Based upon this assumption, we propose that experimentally increasing the “humanness” of others will evoke more vicarious emotions when considering decisions that would harm them, along with increased negative affect resulting from decision conflict. Moreover, there will be a tendency to refrain from such harmful decisions.

Over the past decade, functional Magnetic Resonance Imaging (fMRI) has been frequently applied to study the neural and psychological underpinnings of moral cognition and behavior. By revealing the engagement of areas such as the anterior cingulate cortex (ACC), ventromedial prefrontal cortex (VMPFC), posterior cingulate and posterior temporal cortex, and the anterior insula, these studies have highlighted the importance of domain-general functions for morality, including valuation, affective processing, mental imagery, cognitive control, and social cognition [18], [19], [20], [21], [22], [23], [24], [25], [26], [27], [28], [29], [30], [31], [32]. Assessing activation changes in these areas in response to specific experimental manipulations may provide insights into how certain psychological factors affect the process of decision making under conditions of moral conflict. For instance, comparing “personal” with “impersonal” [18], “difficult” with “easy” [19], and “care-based” with “justice-based” [22], [29] moral dilemmas leads to stronger activations in a subset of the abovementioned brain areas, in particular in medial prefrontal, cingulate and fronto-insular cortex, which has been taken to suggest that these manipulations more strongly tap into affective processes than their supposedly more “cognitively” processed controls [18], [19]. However, others have questioned the plausibility of this “dual track” interpretation, pointing out that complex social deliberation is likely to be mediated by a blend of allegedly emotional-motivational and cognitive processes [33], [34].

Recent attempts to examine the effects of social valuation on moral behavior have shown that judgments about the acceptability of moral violations are affected by whether they involve outgroup members [21] (see also [35]). Sacrificing outgroup members perceived as low in warmth and competence, that is, as supposedly “less human”, was rated as more morally acceptable than sacrificing ingroup members, and saving members of the latter group yielded more activity in medial prefrontal areas. However, using existing outgroup stereotypes (such as drug addicts) to manipulate perceived humanness carries several drawbacks. The potential victims might have differed in many other, potentially confounding aspects, such as assumed hostile or importunate intentions towards others. A further limitation of that study was that the same dilemma was presented in all trials, possibly raising participants’ awareness of the manipulation and thus affecting their responses. Conversely, approaches manipulating the core characteristics of dilemmas by changing the potential “course of action” [18], [19] have the drawback that the effects might be driven by a small number of emotionally salient stimuli having idiosyncratic characteristics or evoking uniform, extreme judgments [36] (but see [37]). Furthermore, the commonly used measure of how “appropriate” or “acceptable” a certain moral violation is judged [18], [19], [20], [21], [24], [27], [28], [30], [31] might tap into different deliberation processes than imagining to be in the depicted dilemma situation and to execute the harmful action oneself. The latter measure might be much closer to probing people’s actual behavioral tendencies and the emotional and cognitive processes underlying them.

In light of these limitations, the aim of the present study was to identify the neural and affective mechanisms by which humanized perception affects moral decision making. To this end, we adopted an experimental approach in which the “perceived humanness” of others was experimentally manipulated in a well-controlled manner – that is, 1) not relying on assumed pre-existing social stereotypes and 2) induced prior to presentation of the moral dilemmas, so that all other characteristics of the moral dilemmas than the “perceived humanness” of the affected person remained unchanged. More specifically, we experimentally humanized the potential victims in dilemma situations using a priming paradigm. During priming, participants were implicitly required to take the affective and cognitive perspective of a fictitious person to trigger more vivid representations of his internal mental states, compared to a neutral control condition where no mentalizing was required. In subsequent moral dilemma situations, participants had to decide about sacrificing the lives of these previously humanized or non-humanized fictitious persons.

We predicted an increased reluctance in taking utilitarian decisions if they involved humanized victims. In addition, and independent of this, we expected the decision process to evoke more vicarious emotions resulting from the anticipated or imagined harm associated with the utilitarian decision. This would trigger increased behavioral conflict, more negative affect and an increased need for regulating these emotions to re-instantiate or maintain utilitarian decision intentions. Thus, we hypothesized 1) that humanized persons would be sacrificed less often to save others in subsequent moral dilemmas; and 2) that decision making involving humanized persons w0uld recruit areas associated with the (prospective) coding of vicarious emotions, negative affect (in particular, anterior insular cortex, anterior midcingulate cortex and VMPFC; e.g. [38], [39], [40], [41], [42], [43]) and with cognitive control and emotion regulation (in particular lateral orbitofrontal and ventrolateral prefrontal areas; e.g. [44], [45], see [46] for a review).

## Materials and Methods

### Participants

We analyzed MRI data from 40 healthy right-handed male volunteers (age 29.2±9.9 years, mean ± standard deviation). Only male volunteers were investigated to increase the homogeneity of our sample. Data from six other participants were discarded because of excessive head-movement (i.e., exceeding 2 mm) during MR scanning, anatomic malformations, or reading problems arising during the scanning session. All subjects had normal or corrected-to-normal vision, and gave written informed consent. The study was performed in accordance with the Declaration of Helsinki and approved by the Ethics Committee of the Medical University of Vienna.

The moral dilemma scenarios were tested in advance in a pilot study involving 74 respondents (49 females; age 36.0±11.1 years, mean ± standard deviation). We also conducted a behavioral experiment as a follow-up to our fMRI experiment. In this experiment we tested 54 male volunteers (age ranging from 19–57, median age 22; none of them had participated in the fMRI experiment).

### Stimulus Material

During the fMRI experiment, participants were presented with blocks consisting of two priming trials and six dilemma trials. In the priming trials, two fictitious persons were described in a short text. This manipulation was akin to priming in the sense that the stimuli, designed to evoke different extents of humanized perception, were assumed to affect responses to dilemma trials presented later on. In these dilemma trials, participants had to decide whether they would sacrifice the life of the primed persons to save the lives of other persons.

#### Priming texts

For priming of the fictitious persons, eight different texts were created. In four of them, young (20–30 years old) male persons were described by repeatedly referring to their mental states (i.e., their thoughts and feelings; Humanized Priming condition). In four other texts, persons with similar appearance and age were introduced without referring to their mental states – i.e., by describing them in a purely factual manner (Neutral Priming condition). The priming manipulation was implicit in the sense that participants were not explicitly instructed about whether or not to take the perspective or to consider the mental states of the fictitious persons.

Above the texts a photograph of the fictitious person’s face was shown, along with a label serving as a name for the person, e.g., “Person A”, “Person B”, etc. Photos were taken from the Radboud Faces Database [47] and depicted frontal shots of young Caucasian males with neutral facial expressions.

Each text was followed by two questions, each with two response options. In the Humanized Priming condition, these questions required the participants to take the perspective of the fictitious person, while in the Neutral Priming condition the questions did not require perspective taking, but instead concerned non-social reasoning, for instance about mechanical devices. In both the Humanized and Neutral condition there was no obvious correct answer to the questions, forcing participants to elaborately consider the information that had been given about the fictitious persons. Hence, questions were typically of the type “why, do you think, is …” or “how, do you think, is …” (see Figure S1 for an example of a Humanized and Figure S2 for an example of a Neutral priming trial; the individuals on the images shown have given written informed consent, as outlined in the PLoS consent form, to publication of their photographs).

#### Moral dilemmas

Twenty-four hypothetical dilemma situations were included in the experiment. Four items were taken from previous work by others and translated into German (three items from the Moral Sense Test by Hauser and colleagues (http://moral.wjh.harvard.edu) and one item from Greene and colleagues [18]. We created 20 additional dilemmas with the following characteristics: 1) a group of people is in danger of dying or suffering serious injury from some external event; 2) if the actor (the participant) does not act, these people will die or be seriously injured; 3) if the actor does act, they can be saved by sacrificing the life of another person who would otherwise stay alive; 4) the participant is not directly involved in the imaginary situation, i.e., he is not in danger himself, nor is he able to sacrifice himself rather than the victim. Crucially, the single person whose life could be sacrificed in the dilemma corresponded to one of the two persons primed before. This was indicated by using the label for that person (e.g. A, B, etc.) in the dilemma text and by showing the photo of that person above the text. Each dilemma text was followed by a question of the type “will you (perform the action)?”, where a “yes” response indicated that participants would engage in the harmful action towards the single person to save the lives of the other persons (utilitarian response), and a “no” response indicated that the participant would spare the primed person and let the other persons die (non-utilitarian response).

We aimed for an average rate of 50% utilitarian decisions in order to maximize the power of the dilemmas in detecting behavioral effects of the priming manipulation. We therefore tested them in advance in a pilot study, without a priming procedure. The six dilemmas yielding less than 25% or more than 75% utilitarian responses were adapted to shift this response proportion towards 50%, for instance by changing the number of persons that were in mortal danger. This resulted in a set of homogeneously difficult dilemma situations. An example of a dilemma situation is given in Figure S3).

#### Post-experimental questionnaire

In order to examine the effects of our manipulation in more detail, we presented the participants with a questionnaire after the scanning session in which we explored their feelings towards the fictitious persons. As a memory aid, participants received a printed version of the priming stories. Using a five-point scale, ranging from 1 (“not at all”) to 5 (“very much”), they had to rate the eight primed persons on the following attributes: (1) “How likable do you find this person?”; (2) “How connected do you feel to this person?”; (3) “How valuable do you find this person?”; (4) “How attractive do you find this person?”; (5) “How annoying do you find this person?” (score reverse-coded); (6) “How well do you understand this person?”; (7) “How well do you feel you know this person?”; and (8) “How similar do you find this person to yourself?”. In addition, we included the following two questions as a manipulation check: (9) “How necessary was it to take this person’s perspective to answer the questions about him?” and (10) “To what extent did your opinion about this person affect your decisions?”.

### Experimental Design and Procedure

#### Design

**Figure 1:**
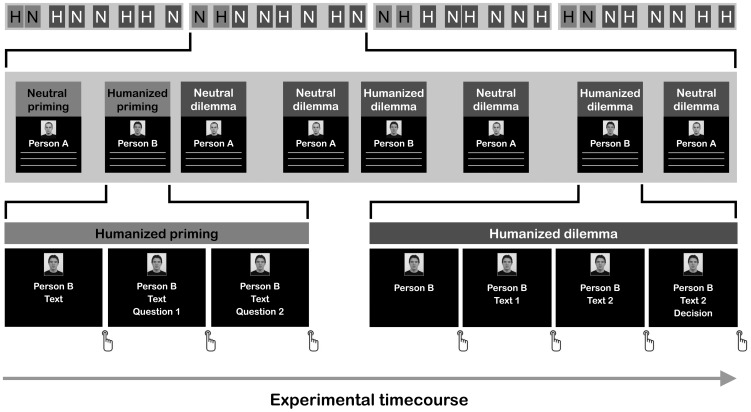
Experimental time course. Top panel: overview of the four blocks constituting the fMRI experiment. Each block started with a Humanized (H) and a Neutral (N) priming trial (this order was pseudorandomized over experimental blocks), followed by three Humanized and three Neutral dilemmas in randomized order. Middle panel: example of a block starting with a Neutral priming trial, in which “Person A” is primed, followed by a Humanized priming trial, priming “Person B”. Each primed fictitious person featured in three of the subsequently presented dilemmas. Bottom panel left: time course of one Humanized priming trial, starting with (1) a screen showing the photo, name, and text describing the fictitious person, followed a by a button press-triggered (2) screen in which a question and two response options were additionally shown; a button response (left or right) triggered (3) a screen in which a second question and two response options replaced the first question; a button response (left or right) ended the trial. Bottom panel right: time course of one Humanized dilemma trial in which “Person B” is included. Each dilemma trial started with (1) a screen showing the photo and the name of the person. After a button press (2) a text describing the emergency situation was added to the screen. A second button press triggered (3) a screen in which the first text was replaced by a second text, describing the respondent’s options to act and the associated consequences for the persons. A new button press added (4) a decision question of the type “will you (perform the action)?” to the screen, along with the response options (“yes” or “no”). A right or left button press ended the dilemma trial. Trials were separated by a variable delay of 3.7–6.9 seconds.

In the experiment, eight priming trials (four Humanized, four Neutral) and 24 dilemma trials (12 Humanized, 12 Neutral) were shown. The trials were presented in four blocks (see Figure 1), each starting with two priming trials (one Humanized, one Neutral) followed by six dilemma trials (three Humanized, three Neutral). The order of the priming trials was pseudo-randomly permuted, so that for each participant 50% of the blocks started with a Humanized and 50% with a Neutral priming trial. In addition, the conditions were randomly permuted over the dilemmas, i.e., the combinations of dilemmas and conditions were varied over participants. Finally, assignment of the portraits to the priming texts was randomly permuted across participants, so that potential effects of the physical appearance of the models on the willingness to harm them were averaged out over participants.

#### Experimental procedures

The experiment was preceded by a 15-min instruction and practice session outside the scanner, in which two separate priming stories and dilemma trials were presented. Participants were instructed to read the priming stories (“person descriptions”) at the beginning of each block carefully and to connect them as well as they could with the photographs. Once they felt they succeeded in doing this, they had to press a button to proceed to the next two screens, on which the two questions were presented. They were told to choose the response option that seemed most appropriate to them. Next, they were informed that in the ensuing “decision situations” one of the persons described before played a decisive role. They were instructed to try as vividly as they could to imagine themselves in these situations, and to answer the ensuing question in a spontaneous, immediate way.

During the scanning session, participants were lying supine in the MR scanner. The stimuli were seen via a back projection system and a mirror that was attached to the head coil. An optical response button box, positioned on the upper right thigh, was used to record subjects’ responses. Stimulus presentation and response recording were carried out using the software package Presentation, version 10.1 (Neurobehavioral Systems, San Francisco, CA). Texts were presented on a black background using white font.

Each of the four blocks started with two priming trials (see Figure 1). Priming trials started with a black screen showing a photo, a name (e.g. “Person A”), and a text describing the fictitious person. Participants could proceed to the next screen by pressing a button with their right index finger. On the next screen, along with the previous contents, a question and two response options were displayed. The two response options were displayed at the left and right bottom side of the screen. After responding to the question with either a corresponding left (index finger) or right (middle finger) button press, the next question was shown. Responding to this question ended the trial.

After two priming trials, six dilemma trials were presented. Each dilemma trial consisted of the following four parts. First, a screen with the photograph of one of the primed persons and his name was shown. Participants had been instructed to actively recall who this person was before pressing a button to trigger the next screen. On the next screen, a text describing an emergency situation was added to the photo and name information. After reading this text, another button press initiated a third screen in which the first text was replaced by a second text, describing the participant’s options to act and the associated consequences for the primed person and the other persons involved in the emergency. With a further button press a question of the type “Will you (perform the action)?” was added to the screen, along with the response options (“yes” or “no”). An index finger response corresponded to a “yes” (i.e., a utilitarian) response, and a middle finger response to a “no” (i.e., a non-utilitarian) response. By responding, participants ended the dilemma.

All trials were separated by a variable intertrial interval of 3.7–6.9 seconds, in which a black screen was shown. Total duration of the experiment depended on individual reading speed and varied from 24 to 58 minutes, with an average of 36 minutes. After the scanning session, participants were asked to fill out the post-experimental questionnaire about the primed persons.

### Analysis of Behavioral Data

During the experiment, both the button responses to proceed to the next screen and the responses to the priming and dilemmas questions were recorded. The timing of these button presses was used to create the regressors modeling the different experimental epochs (see below, Imaging analysis).

Responses to the dilemmas obtained during the scanning session and post-scanning ratings of the primed fictitious persons were analyzed using PASW 18.0 (SPSS Inc., Chicago, IL, USA). The effect of Priming condition (Humanized, Neutral) on moral decisions was assessed by comparing the number of utilitarian decisions affecting Humanized vs. Neutral persons using a paired t-test. Likewise, the main effect of Priming condition (Humanized, Neutral) on the post-experimental ratings was assessed using a paired t-test on the overall sum rating of items 1–8 (the “connectedness” items), as well as on items 9–10 and on all individual items, using Bonferroni-correction for multiple comparisons. Alpha level was set to 0.05 for all analyses of behavioral data.

### MRI Acquisition

MRI data were acquired using a 3 Tesla Siemens Tim Trio MRI system (Siemens Medical, Erlangen, Germany) using a 32-channel head coil for signal reception. Blood oxygen level-dependent (BOLD) sensitive functional imaging was performed using an echoplanar imaging (EPI) sequence with Generalized Autocalibrating Partially Parallel Acquisition (GRAPPA) and the following parameters: echo time (TE)/repetition time (TR) = 40/2000 ms, flip angle 90°, interleaved acquisition, 25 axial slices co-planar the connecting line between anterior and posterior commissure, FOV 210×210 mm, matrix size 128×128, voxel size 1.64×1.64×4.75 mm, interslice gap 2.85 mm). Structural images were acquired after functional scanning using a magnetization-prepared rapid gradient-echo (MPRAGE) sequence (TE/TR = 4.21/2300 ms, 160 sagittal slices, voxel size = 1.0×1.0×1.1 mm, field of view = 256 mm).

### Analysis of MRI Data

MRI data were analyzed using SPM8 (Statistical Parametric Mapping, http://www.fil.ion.ucl.ac.uk/spm). The first five volumes of each participant’s fMRI data were discarded to allow for T1 equilibration. The time series for each voxel was then realigned temporally to the acquisition of the first slice in time to correct for differences in slice time acquisition [48]. The image time series were spatially realigned using a sinc interpolation algorithm that estimates rigid body transformations (translations, rotations) by minimizing head-movements between each image and the reference image [49]. Subsequently, each participant’s structural image was segmented into gray matter (GM), white matter (WM), and cerebral spinal fluid (CSF) using GM, WM, and CSF tissue probability maps provided by SPM8 and then spatially normalized to the International Consortium for Brain Mapping (ICBM) space templates (European brains) using both linear and nonlinear transformations. The participant’s functional images were then spatially coregistered to the non-normalized structural image and spatially normalized by using the same transformation matrix as applied to the structural image. The brain extraction tool (BET) of FSL (FMRIB Software Library, http://www.fmrib.ox.ac.uk/fsl) was used to remove non-brain tissue from the images. Finally, the images were spatially smoothed using an isotropic 8 mm full-width-at-half-maximum Gaussian kernel.

The fMRI time series were analyzed using an event-related approach in the context of the General Linear Model (GLM). Trial-by-trial measures of the timing of the different epochs within each trial were extracted from the button responses collected during the experiment. Single-subject models consisted of multiple regressors describing, separately for Humanized and Neutral trials, 1) the reading period of the priming trials, 2) the question period of the priming trials (i.e., the period in which the questions had to be read and answered), 3) the first stages of the dilemma trials (i.e., the period in which the photo and name, and subsequently the first text were shown), 4) the second stage of the dilemma trials (i.e., the period in which the second text was presented), and finally 5) the dilemma question phase (i.e., the period in which the dilemma question was presented on the screen, until a response was made).

Each effect was modeled on a trial-by-trial basis as a concatenation of square-wave functions. Each of these square-wave functions was then convolved with a canonical hemodynamic response function, as implemented in SPM8, in order to generate 10 regressors modeling the main effects described above [49].

Head movement effects were accounted for by including the six rigid-body motion parameters (translation and rotation) as well as two regressors describing intensities in white matter (WM) and cerebrospinal fluid (CSF) as nuisance covariates. We used MarsBaR (http://www.marsbar.sourceforge.net) to extract the scan-by-scan signal in individual WM and CSF masks.

### Statistical Inference

The statistical significance of the estimated evoked hemodynamic responses was assessed using t-statistics in the context of general linear model-based analyses, as implemented in SPM8. We were specifically interested in assessing effects of our priming manipulation (Humanized, Neutral) on brain activity during the priming question and dilemma question stages, since we expected these phases to be most sensitive to the effects of our manipulation. In the priming trials, the last phase reflected the period in which participants tried to answer the question, requiring either mentalizing (Humanized condition) or not (Neutral condition). In the dilemma trials, the last phase comprised the period in which participants had to decide about whether they would sacrifice the life of the Humanized or Neutral primed person to save the lives of the others in the hypothetical situation.

For this purpose, contrasts of the parameter estimates for the question periods of the priming and dilemmas trials were calculated, separately for Humanized (i.e., Humanized Priming > baseline, Humanized Dilemmas > baseline) and Neutral trials (i.e., Neutral Priming > baseline, Neutral Dilemmas > baseline), where the intertrial interval was considered the implicitly modeled baseline. These contrasts were entered into a random-effects second-level analysis using paired t-tests (Humanized > Neutral Priming and the reverse contrast, and Humanized > Neutral Dilemmas and the reverse contrast), in order to enable inferences on a population level [50].

Statistical inference was performed using a threshold of P = 0.05 corrected for multiple comparisons over the whole brain, using the Gaussian random fields approach at cluster-level with a voxel-level intensity threshold of P = 0.005 [51]. The SPM Anatomy Toolbox [52] was used to guide anatomical and probabilistic cytoarchitectonic localization of the resulting clusters.

### Effective Connectivity Analysis

In order to substantiate our interpretation of the main effects, we performed an exploratory connectivity analysis, testing for regions whose coupling with a region in the right anterior midcingulate cortex (aMCC) associated with conflict monitoring and behavioral control was modulated as a function of humanization. To this end, we used the psychophysiological interactions (PPI) method [53] to search for areas which had a higher correlation with the time-course in the aMCC during Humanized than during Neutral dilemma decision phases. For each participant, we extracted the first eigenvariate of the time series of all voxels (i.e., the physiological activity) within a spherical VOI with a radius of 5 mm around coordinate (2 13 47). This coordinate was located within a broader significant cluster in the main analysis and corresponded to a region showing overlapping responses to negative affect, pain and cognitive control, as observed in a recent coordinate-based meta-analysis [43]. Hence, the seed region extended dorsally from the aMCC and also included parts of the rostral cingulate zone (RCZ) and the supplementary motor area (SMA). The PPI regressor was obtained by multiplying the estimated physiological activity from the seed region with a vector coding for the time course of our experimental task (1 for Humanized Dilemma decisions, −1 for Neutral Dilemma decisions). Then for each participant a GLM whole-brain analysis was performed, with the PPI regressor, the experimental contrast (Humanized versus Neutral dilemma decisions), and the estimated neuronal activity from the seed region as predictor variables. For each participant, contrast images were generated for the PPI regressor, which were then entered into a one-sample t-test at the second (group) level. Inferences were corrected for multiple comparisons (corrected threshold p<0.05, given an intensity threshold of p<0.001 [51]; we used a more stringent intensity threshold than the a priori selected threshold of 0.005 to achieve a more precise localization).

### Follow-up Behavioral Experiment on Decision-related Affective and Cognitive Processes

To further clarify the affective and cognitive processes involved in moral decision making, as measured with our newly developed paradigm, we conducted an additional behavioral experiment. In this experiment, the participants completed a shortened version of the fMRI experiment. Seated in front of a computer screen in a soundproof room, they were first presented with two blocks of two priming trials and two dilemma trials each, requiring four dilemma decisions (two Humanized, two Neutral).

In the second part of the experiment, the participants were asked to recall the decision phase of each dilemma and rate the emotions they had experienced during this phase. As a memory aid, the photographs of the primed persons along with some keywords referring to the priming text and the respective dilemma in which the person had been involved were shown on the screen. A digital visual analogue scale was used to collect the ratings, consisting of a slider that had to be positioned between the two extremes “not at all” and “very much”, referring to the respective emotion felt when making the decision. Responses were collected on three subscales, of which two measured the level of Personal Distress and Empathic Concern as two distinct vicarious emotions experienced in response to the distress of others [54], [55]. The third subscale (Humanization) was created to assess the extent and the characteristics of the humanization effect. Each subscale contained three items; the resulting nine items were presented in randomized order. More specifically, the Personal Distress scale assessed self-oriented aversive emotions with questions about how frustrated, torn or stressed participants felt during the decision. The Empathic Concern scale assessed other-oriented emotions with the items “How strongly did you imagine the thoughts and feelings of this person?”, “How much compassion did you feel for this person?”, and “How moved were you during the decision moment?”. The Humanization subscale assessed the effects induced by the priming manipulation using the questions: “To what extent did you see this person as a human being, rather than a means to an end?”, “How responsible did you feel for this person’s well-being?”, and “To what extent did you see this person as a human being with needs, desires, and feelings? The intention of this subscale was to assess the consequences of humanization in terms of the key characteristics of perceiving someone as human (being more than just an “object” to achieve a goal, inducing a sense of responsibility, and having mental representations which non-human entities do not have). Internal consistency of this subscale was acceptable, as was assessed post hoc (Cronbach’s alpha = 0.72).

The final part of the behavioral experiment was designed to further examine the effects of the humanization manipulation. Participants were asked to rate each of the four primed persons on a number of characteristics, which constituted two subscales. The first subscale was intended to measure “Interpersonal Connectedness” and consisted of items 2,6,7,8 from the questionnaire of the fMRI study. Grouping these items together was justified by their high internal consistency on the post-experimental questionnaire. (Cronbach’s alpha = o.84). The second subscale consisted of five newly developed items: “How 1) alive, 2) tangible, 3) human, 4) abstract, and 5) interchangeable (with another person) did this person seem to you?” The responses to these questions were summed up (with the “abstract” and "interchangeable” items being reverse-coded) to calculate an index of the “Perceived Humanness” of the victim. Internal consistency of this scale was acceptable, as was assessed post hoc (Cronbach’s alpha = 0.72). All items were presented in randomly permuted order. The duration of the follow-up behavioral experiment was 35–45 minutes, depending on individual reading speed and response times.

The aggregated ratings of the dilemma decision were included in a 2×3 repeated-measures ANOVA with factors Priming condition (Humanized, Neutral) and Rating scale (Personal Distress, Empathic Concern, Humanization). Planned comparisons and post-hoc t-tests were applied to assess specific effects in more detail. Finally, the ratings of the primed fictitious persons on the Interpersonal Connectedness scale and Perceived Humanness scale were analyzed using paired t-tests.

## Results

### Behavioral Results

**Figure 2:**
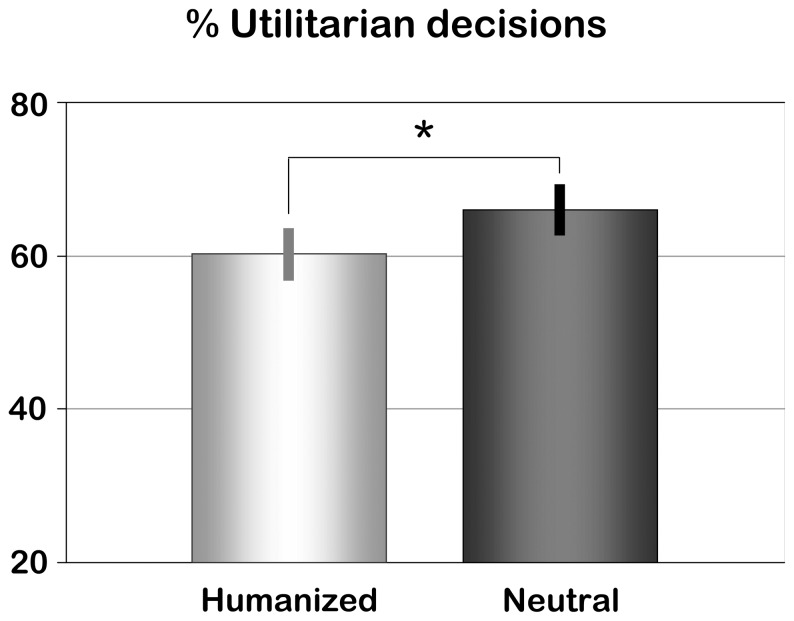
Dilemma decisions. Percentage of utilitarian decisions (i.e., single person is sacrificed to save numerous others threatened by imminent death or injury) in dilemmas involving Humanized or Neutral persons. Error bars denote standard errors of the mean.

Participants made significantly fewer utilitarian decisions when the dilemma involved a humanized person (mean (M) = 60.2%, standard error (SE) = 3.3) than when it involved a neutral person (M = 66.0%, SE = 3.4; see Figure 2), confirming that the priming manipulation yielded the predicted effects (paired t-test, T(39) = 2.27, p = 0.029). Mean response time to the dilemmas was 5.2 seconds (SE = 0.48). Response times were not affected by factors Decision (Utilitarian, Non-utilitarian; F(33) = 1.74, p = 0.20), Priming (Humanized, Neutral; F(33) = 0.12, p = 0.73), or their interaction (F(33) = 0.27, p = 0.61; note that this analysis was performed on a subset of 34 participants, since not all participants had made both types of decisions for both conditions).

The combined ratings on the post-fMRI questionnaire measuring affect towards the primed persons were significantly higher for humanized (M = 3.06, SE = 0.06) than for neutral (M = 2.82, SE = 0.05) persons (paired t-test, T(39) = 3.83 p<0.001). Post-hoc paired t-tests on all 10 items (Bonferroni-corrected, α = 0.005) revealed that this effect was mainly driven by differential ratings on the items “How connected do you feel to this person?” (T(39) = 3.42, p = 0.001); “How well do you understand this person?” (T(39) = 3.48, p = 0.001); “How well do you feel you know this person?” (T(39) = 4.00, p<0.001); and “How similar do you find this person to yourself?” (T(39) = 5.47, p<0.001). Notably, no significant difference was observed for the question “How likable do you find this person?” (T(39) = 1.64, p = 0.11; Humanized: M = 3.36, SE = 0.09; Neutral: M = 3.18 SE = 0.08).

The emotion ratings about the dilemma decision phase obtained in the follow-up behavioral experiment, which had been recoded to a continuous scale ranging from 0 to 100, were included in a 2×3 repeated-measures ANOVA with factors priming condition (Humanized, Neutral) and rating scale (Personal Distress (PD), Empathic Concern (EC), Humanization (H)). There was a significant main effect of Priming condition (Humanized, Neutral) on ratings averaged across all items (Humanized: M = 58.9, SE = 2.4; Neutral: M = 55.4, SE = 2.1, F(53) = 4.59, p = 0.037), but no effect of rating scale (PD, EC, H; F(53) = 1.21, p = 0.297, Greenhouse-Geisser corrected) and no interaction between those factors (F(53) = 0.39, p = 0.628, Greenhouse-Geisser corrected). A priori planned comparisons using one-tailed paired t-tests revealed significantly higher scores for dilemmas with humanized than neutral persons on the H scale (Humanized: M = 60.7, SE = 2,5, Neutral: M = 56.2, SE = 2.5, T(53) = 2.36, p = 0.011), a strong trend towards higher scores on the PD scale for dilemmas with Humanized persons (Humanized: M = 59.5, SE = 3.3, Neutral: M = 56.0, SE = 2.6, T(53) = 1.63, p = 0.055), and a weaker trend towards higher scores on the EC scale (Humanized: M = 56.4, SE = 2,5, Neutral: M = 53.8, SE = 2.5, T(53) = 1.30, p = 0.099). Notably, a direct comparison of the effects of Priming condition on PD and EC ratings revealed no significant difference between the two types of vicarious emotions (T(53) = 0.40, p = 0.69). This motivated a post-hoc paired t-test of aggregate PD and EC scores, which showed that humanized dilemmas resulted in higher values of this combined measure of vicarious emotions (Humanized: M = 58.0, SE = 3.3, Neutral: M = 54.9, SE = 1.7, T(53) = 1.77, p = 0.041).

Ratings of the primed persons on scales of Perceived Humanness (PH) and feelings of Interpersonal Connectedness (IC) showed a highly significant effect of the priming manipulation, with higher ratings for Humanized than Neutral persons on both the PH scale (Humanized: M = 61.4, SE = 1.9, Neutral: M = 56.9, SE = 2.0, T(53) = 2.20, p = 0.016) and the IC scale (Humanized: M = 50.9, SE = 2.3, Neutral: M = 43.5, SE = 1.8, T(53) = 2.20, p = 0.016).

### fMRI Results

#### Priming of humanized versus neutral persons

**Figure 3:**
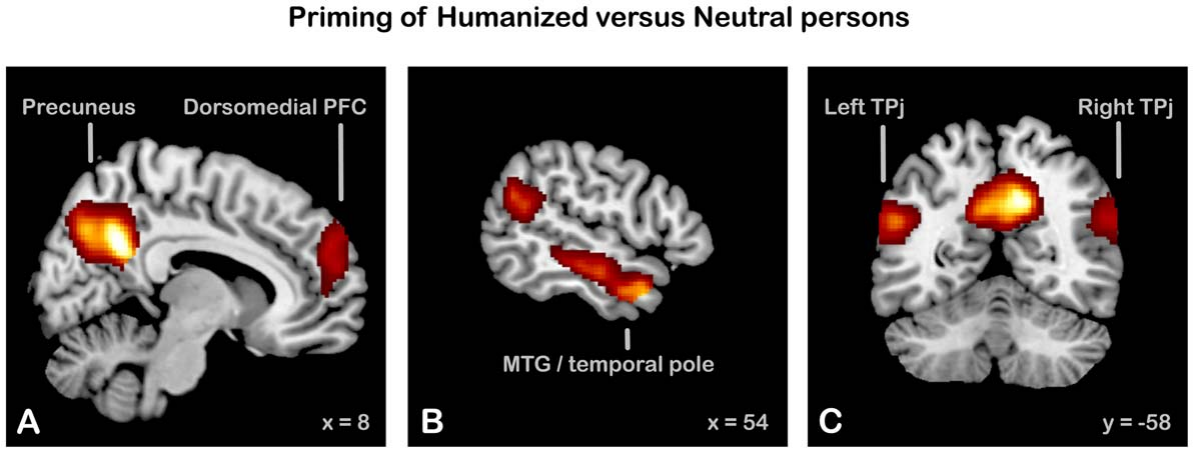
Statistical parametric maps (SPMs) showing increased activity during the question phase of Humanized (H) as compared to Neutral (N) priming trials, requiring either mentalizing (H) or not (N). (A) bilateral precuneus/PCC and dorsomedial prefrontal cortex (PFC) (B) right middle temporal gyrus (MTG)/temporal pole; a cluster of comparable size was also present in the left hemisphere (C) bilateral temporo-parietal junction (TPJ). SPMs are displayed in neurological convention on the high-resolution structural MRI template brain provided in SPM8, threshold P = 0.05, corrected for multiple comparisons at the cluster-level.

The contrast Humanized > Neutral Priming revealed an extensive network of brain areas (see Table 1, Figure 3) that included a large bilateral cluster in the precuneus/posterior cingulate cortex (PCC), two clusters extending from the middle temporal gyrus (MTG) to the temporal poles bilaterally, two clusters at the intersection of temporal and parietal cortex in the left and right hemisphere, corresponding to an area referred to as the temporo-parietal junction (TPJ [56]), a bilateral cluster in the dorsal middle frontal gyrus (dorsomedial prefrontal cortex, dmPFC), and two clusters in the left and right calcarine sulcus. The reverse comparison (Neutral > Humanized priming) yielded a network of mainly superior parietal and dorsal prefrontal areas.

**Table 1:** MNI stereotactic coordinates of the local maxima of the activation clusters resulting from the contrasts assessing differences between answering questions related to Humanized versus Neutral fictitious persons (top), and between deciding about dilemmas involving Humanized versus Neutral persons (bottom).

Area	Hemisphere	PeakMNI-coordinates			Cluster size(voxels)	T-Value	P-value (corrected)
		x	y	z			
**Priming questions about Humanized > Neutral persons**							
Precuneus/PCC	Left + Right	8	−54	32	3876	11.92	<0.001
MTG/temporal pole	Right	50	12	−28	1663	9.39	<0.001
TPj	Left	−52	−58	24	772	7.44	0.002
MTG/temporal pole	Left	−50	−30	−8	956	6.62	0.001
TPj	Right	54	−56	26	803	6.59	0.002
dmPFC	Left + Right	6	52	26	1231	6.07	<0.001
Calcarine sulcus	Right	16	−94	2	636	5.99	0.007
Calcarine sulcus	Left	−14	−98	−2	481	5.26	0.027
**Dilemma decisions about Humanized > Neutral persons**							
pgACC/VMPFC/mOFC	Left + Right	6	40	−2	1249	5.26	<0.001
Anterior insula/IFG (pars opercularis – VLPFC)	Right	42	10	14	905	4.86	0.002
Anterior insula/IFG (pars orbitalis - OFC)	Left	−22	28	−14	431	4.56	0.069
Precuneus/PCC	Left + Right	−6	−40	40	1092	4.51	0.001
SMA	Left + Right	−12	2	68	1269	4.50	<0.001
MTG/temporal pole/posterior insula	Right	38	−24	4	658	4.35	0.011
aMCC	Left + Right	8	16	30	581	3.90	0.020

PCC = posterior cingulate cortex; MTG = middle temporal gyrus; TPJ = temporo-parietal junction; dmPFC = dorsomedial prefrontal cortex; pgACC = perigenual anterior cingulate cortex; VMPFC = ventromedial prefrontal cortex; mOFC = medial orbitofrontal cortex; IFG = inferior frontal gyrus; VLPFC = ventrolateral prefrontal cortex; SMA = supplementary motor area; aMCC = anterior midcingulate cortex.

#### Dilemma decisions about humanized versus neutral persons

**Figure 4:**
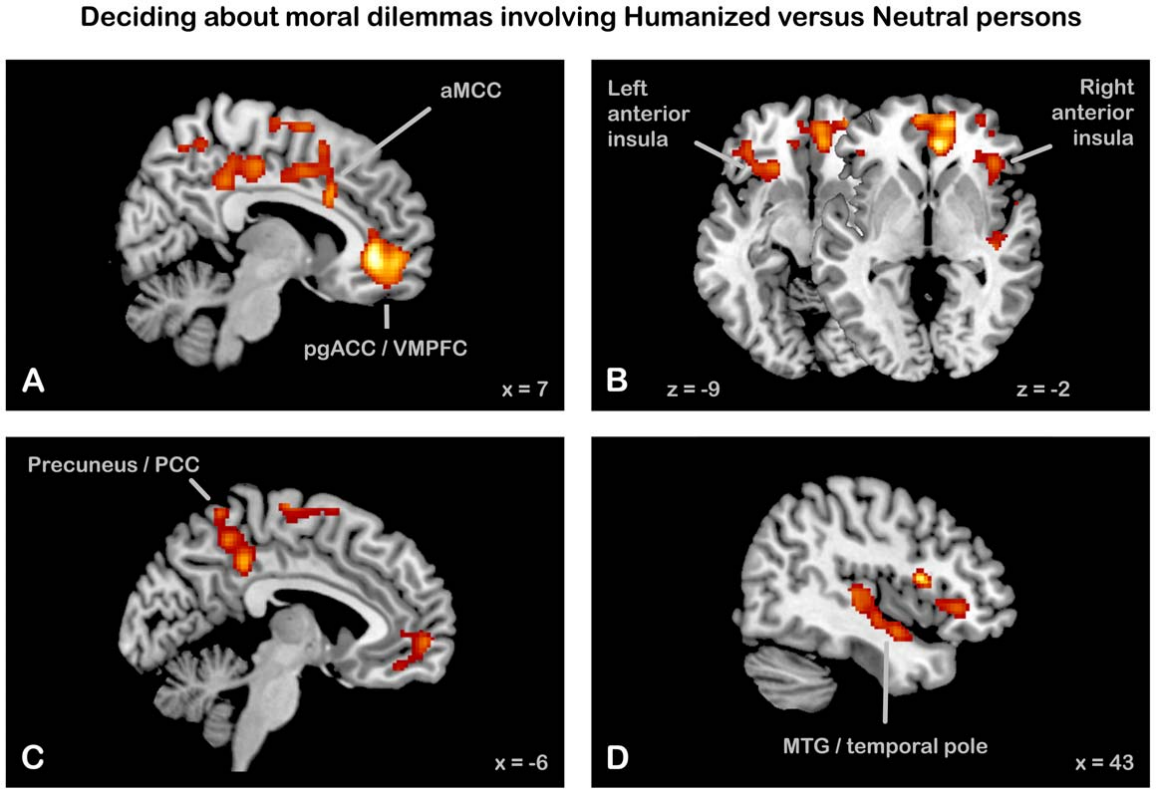
SPMs showing increased activity for the contrast Humanized > Neutral during moral dilemma decisions. (A) bilateral perigenual anterior cingulate cortex (pgACC)/ventromedial prefrontal cortex (VLPFC)/medial orbitofrontal cortex (mOFG) and right anterior midcingulate cortex (aMCC) (B) bilateral anterior insula/inferior frontal gyrus (IFG) (C) bilateral precuneus/posterior cingulated cortex (PCC) (D) right middle temporal gyrus (MTG)/temporal pole extending into posterior insula. See Figure 3 for other specifications.

The comparison between dilemma decisions involving Humanized versus Neutral persons (contrast Humanized > Neutral Dilemmas) revealed the following significant clusters (see Table 1, Figure 4, for details). First, a large cluster with its maximum in right perigenual anterior cingulate cortex (pgACC), extending bilaterally into the mid-orbital gyrus and rightward also into the superior orbital gyrus. Thus, this cluster covered sub- and perigenual ACC and ventromedial prefrontal cortex (VMPFC)/medial orbitofrontal cortex (mOFC). Second, we observed activations in the dorsal part of the right anterior insula, extending into the pars opercularis of the inferior frontal gyrus (IFG; corresponding to the ventrolateral prefrontal cortex (VLPFC)), and in the ventral part of the left anterior insula, extending into the pars orbitalis of the IFG. The latter cluster did not survive the FWE-corrected threshold of 0.05, but showed a trend towards significance (p(corrected) = 0.069). Third, significant clusters were revealed in bilateral precuneus/PCC, in bilateral rostral supplementary motor area (SMA), and in right temporal cortex extending along the MTG towards the temporal pole and also including posterior parts of the insula. Finally, a cluster in the cingulate cortex was detected whose peak coordinate was located in the anterior part of the midcingulate cortex (aMCC [57]) but which caudally extended into posterior MCC and dorsally into an area referred to as rostral cingulate zone (RCZ [43], [58]). Testing for the reverse contrast, i.e., comparing dilemma decisions about Neutral with Humanized persons, did not yield any clusters above threshold.

#### Modulations of aMCC connectivity by humanized versus neutral dilemma decisions

**Figure 5:**
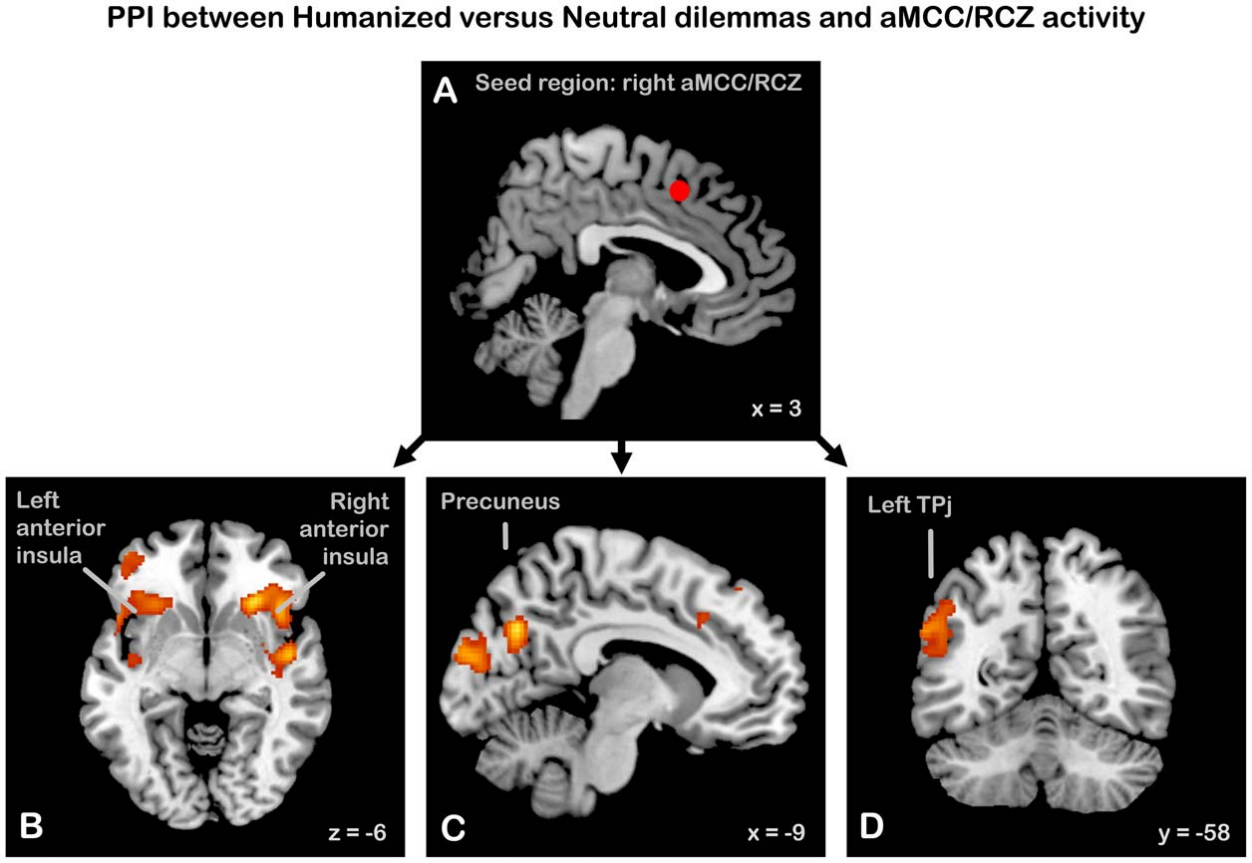
Areas showing increased coupling with right anterior midcingulate cortex (aMCC)/rostral cingulate zone (RCZ) during dilemma decisions involving Humanized versus Neutral persons. (A) The seed region in aMCC/RCZ, defined by a sphere with a radius of 5 mm around (2 13 47). (B) Bilateral frontoinsular cortex (C) Bilateral precuneus and cuneus (D) Left temporo-parietal junction (TPJ). Other specifications as in Figure 3.

The PPI analysis testing for regions that showed increased coupling with aMCC/RCZ during Humanized compared to Neutral dilemmas identified a set of regions that involved among others bilateral (mainly left) precuneus extending into the cuneus, extensive clusters bilaterally covering the anterior insula, extending into adjacent IFG (pars triangularis and orbitalis) and on the left side into the middle orbital gyrus, and a cluster in the left angular gyrus/TPJ (see Table 2 and Figure 5 for details).

**Table 2:** MNI stereotactic coordinates of the local maxima of the activation clusters resulting from the PPI analysis testing for increased coupling with aMCC/RCZ during dilemma decisions about Humanized versus Neutral persons.

Area	Hemisphere	PeakMNI-coordinates			Cluster size	T-Value	P-value (corrected)
		x	y	z			
**Increased effective connectivity with aMCC/RCZ during dilemma decisions about Humanized > Neutral persons**							
Precuneus/Cuneus	Left + Right	68	−54	32	1223	4.94	<0.001
Anterior insula/IFG	Right	28	24	−6	1227	4.85	<0.001
Postcentral gyrus (S1)	Left	−44	−28	48	625	4.69	0.006
Posterior insula	Right	36	−22	4	643	4.67	0.005
Anterior insula/IFG	Left	−54	8	2	2044	4.43	<0.001
Angular gyrus (TPJ)	Left	−58	−58	28	338	4.16	0.049
aMCC/RCZ	Left + Right	4	22	24	761	4.01	0.002

IFG = inferior frontal gyrus; S1 = primary somatosensory cortex; TPJ = temporo-parietal junction; aMCC = anterior midcingulate cortex; RCZ = rostral cingulate zone.

## Discussion

The aim of our study was to investigate the effects of experimentally “humanized” potential victims on behavioral, affective and neural responses during moral decision making. We used an implicit priming paradigm that required participants to either engage in mentalizing with primed fictitious persons (Humanized condition) or not (Neutral condition). We hypothesized that humanized persons would be sacrificed less often to save others in subsequent moral dilemmas, and that decision making in dilemma situations involving humanized victims w0uld increase involvement of areas associated with coding vicarious emotions, behavioral conflict, negative affect, as well as with regulating those emotional responses. Our results largely confirm these hypotheses. In the following sections, we discuss our findings in detail and elaborate on their implications for understanding the role of humanized perception in decision making and prosocial behavior.

### Brain Activity during Humanization

The priming manipulation, designed to increase the perceived humanness of the fictitious persons in the Humanized condition, evoked strong bilateral activity in dorsomedial prefrontal cortex, precuneus/PCC, MTG/temporopolar cortex, and TPJ. This network of brain areas has been consistently associated with mentalizing and theory of mind [4], [59], [60], [61], [62], [63], [64]. Hence, these results confirm that our manipulation was effective in evoking mentalizing processes during humanized priming, in contrast to neutral priming. That is, when answering humanizing questions as compared to neutral questions participants indeed seemed more strongly engaged in reflections about the mental states of these persons.

### Behavioral Effects of Humanization

Deciding about dilemmas involving humanized versus neutrally primed persons was associated with a lower proportion of utilitarian decisions. That is, in the fMRI-experiment, humanized persons were sacrificed less often than neutral persons in moral dilemma situations. Note, though, that the difference was fairly small (60.2% versus 66.0% utilitarian responses in Humanized versus Neutral dilemmas, respectively, corresponding to an effect size Cohen’s *d* = 0.36), and that the majority of decisions affecting humanized persons still was utilitarian. This indicates that participants were generally able to overcome potential behavioral and emotional conflict associated with decisions affecting these persons.

In post-fMRI ratings participants reported that they felt more similar and connected to humanized persons and felt to understand and know them better than neutral persons. Humanized persons were not rated as more likable, though, suggesting that the effects on decision making cannot simply be explained by differences in liking, although the fact that there was a trend (p = 0.1) in this direction does not exclude the possibility that humanization affects liking to some degree. In the follow-up behavioral experiment, we replicated the finding that humanized persons evoked increased feelings of “Interpersonal Connectedness”, and in addition assessed to what extent the primed persons were more strongly perceived as “human”. The “Perceived Humanness” scale included ratings of how “alive”, “tangible”, “human”, “abstract” (rating reversed) and “interchangeable” (rating reversed) the primed persons seemed to the participants. Humanized persons received higher ratings on this scale, further confirming the efficacy of our manipulation. Additionally, in this post-hoc experiment participants’ ratings of their feelings *during* the decision process indicated that they saw Humanized persons more strongly “as a human being, rather than a means to an end”, more strongly conceived of them as “a human being with needs, desires, and feelings”, and notably also felt more “responsible for the person’s well-being”. This not only indicates that participants had a more vivid representation of the human characteristics of the humanized persons, but also that this was relevant for potential actions towards these persons.

In the follow-up behavioral experiment we also assessed ratings on two classes of vicarious emotions: personal distress (PD) and empathic concern (EC), with the first scale targeting self-related emotions and the latter other-related emotions triggered by the confrontation with another person in distress [54]. We hypothesized that humanized persons would evoke stronger vicarious responses, and also explored whether those emotions were rather self- or other-related. The latter distinction is important, as it has been theorized that while self-related emotions lead to withdrawal, other-related emotions result in an increase in prosocial motivation and possibly helping behavior (e.g. [65]). Combined ratings on these scales were higher for decisions about humanized than neutral persons. Testing for effects of humanization on the two scales separately showed that effects on PD were just above significance threshold, and that there was a trend for EC. However, the difference scores between ratings of humanized and neutral dilemmas did not differ for PD and EC. Thus, moral decision making about humanized persons elicits stronger vicarious emotions, with self- or other-related emotions occurring to a similar extent.

### Brain Activity Related to Moral Decision making about Humanized Persons

Deciding about dilemmas involving humanized persons evoked increased activity in bilateral pgACC extending into VMPFC and mOFC; bilateral anterior insula extending into the IFG (right VLPFC and left lateral OFC); bilateral precuneus/PCC; bilateral SMA; right MTG/temporopolar cortex extending into posterior insula; and aMCC extending dorsally into the RCZ and SMA, and caudally into pMCC.

This set of brain areas is largely consistent with previous findings on neural responses to stimuli with high moral significance (see [66] for a comprehensive review). More specifically, two studies contrasting “personal” with “impersonal” dilemmas, where “personal” denotes a situation in which the harmful act is relatively direct, “authored”, and aimed towards a specific person, found increased contributions of mPFC, precuneus/PCC, midtemporal and temporoparietal cortex, and MCC [18], [19]. The same authors also contrasted “difficult” with “easy” moral dilemmas, which led to increased contributions of ACC, DLPFC, anterior insula, and precuneus/PCC. A study contrasting “care-based” dilemmas (appealing to social emotions) with “justice-based” dilemmas (appealing to rules and obligations), found increases in PCC, VMPFC and DLPFC [29]. Notably, all these studies used manipulations that might have increased the perceived humanness of the persons involved in the scenarios. In the following paragraphs we discuss the functions of the areas recruited by our manipulation in more detail and draw inferences on the nature of the humanization effect.

#### pgACC/VMPFC/mOFC

The activation cluster in pgACC/VMPFC/mOFC is highly consistent with the frequently observed involvement of this area in viewing or judging stimuli with high moral conflict (e.g. [24], [27], [29], [30]). Lesion studies indicate that the VMPFC seems essential for “feeling bad” about moral violations [12], [39], [67], [68], [69], [70], in line with the more general notion that this area is involved in the affective evaluation (“valuation”) of response options. That is, VMPFC and adjacent pgACC seem to evaluate the reward value of (actual or predicted) states and to signal aversive or conflicting situations requiring increased behavioral control [38], [46], [57], [69], [71], [72], [73]. Some have suggested that this area is also involved in emotion regulation [44], [68], [74], [75], but this view is not undisputed [46], [69].

#### Anterior insula/IFG and aMCC

The anterior insula, along with parts of adjacent IFG, is associated with generating a global, subjective representation of the current or predicted bodily state (e.g. [72], [76]). It is commonly activated in aversive situations, involving fear, disgust or pain, both when involving oneself and when observing others who are suffering [42], [55], [71], [77]. Thus, the anterior insula is strongly involved in feelings of empathy and other vicarious emotions, both when the affective states of others are actually observed and when they are merely anticipated or imagined (see [10], [40], [41], [78] for reviews).

The aMCC and the anterior insula are heavily interconnected [79], [80], and the anterior insula is often co-activated with the aMCC during social interaction [42], [55], [77], as was also observed in the present study. The aMCC and adjacent parts of the RCZ seem to play a role in signaling pain, punishment, and aversive states, but also in increasing performance monitoring, as in situations with high uncertainty or conflict about the optimal behavioral response (see [46], [57] for reviews). According to a recent account, these processes cannot be functionally segregated: both negative affect and increased cognitive control might be seen as part of an “early warning system” calling for behavioral, physiological and cognitive adaptations. Thus, the aMCC might regulate behavioral responses by forming a hub between negatively valuated information and motor centers [43].

#### Precuneus/PCC

The humanization manipulation also evoked activity in precuneus/PCC during moral decision making. The precuneus/PCC has been linked to generating subjective experiences from emotions [72], visual and memory-based imagery [59], [81], self-referential thought [23], [82], empathy [25], [83], [84], [85], mentalizing [64], and perspective taking [85], [86]. This area is also recruited more strongly during difficult (versus easy [18]), “personal” (versus “impersonal” [18], [19]), care-based [29], or emotionally salient [24] moral scenarios.

#### Implications of the imaging findings for understanding the role of humanized perception in moral decision making

Taken together, deciding about moral dilemmas involving humanized rather than neutrally primed persons recruits a network of brain areas involved in negative affect, emotional imagery, empathy and signaling aversive or conflicting situations, but also in emotion regulation and behavioral control (e.g., via response inhibition). The picture emerging from this is that deciding about dilemmas in which the life of a single humanized persons has to be weighed against the lives of several other people denotes a more conflicting situation, evoking increased self-related aversive reactions, and increased feelings of empathic concern with the primed person. These negative emotions might require increased regulatory and reappraisal processes to enable quick and “rational” decision making – although despite these presumed regulatory efforts, our participants still made slightly fewer utilitarian decisions when humanized victims were involved. Importantly, this interpretation is supported by our behavioral follow-up experiment, which showed that decisions about humanized persons evoked stronger vicarious emotions than decisions about neutral persons.

#### Effective connectivity of aMCC

Our interpretation receives additional support from the effective connectivity analysis which tested for regions increasing their coupling to the right aMCC/RCZ during moral decision making involving humanized persons. The PPI analysis showed that the aMCC/RCZ increased its coupling with bilateral anterior insula, precuneus and adjoining cuneus, and left TPJ during decisions in Humanized dilemmas (see Figure 5). The increased effective coupling between aMCC and anterior insula suggests an increase in information flow between these areas during dilemmas involving humanized persons. This might reflect increased signaling of negatively experienced motivational states (reflecting response conflict) by the anterior insula to the aMCC, to aid in heightened control for selecting the optimal course of action [43], [87]. Thus, the selective increase in connectivity between these areas during moral decisions about humanized persons is congruent with our interpretation that these decisions constitute a more conflicting situation.

The aMCC/RCZ also increased its coupling with the precuneus and cuneus, and with the left TPJ. In line with the known involvement of both the precuneus and the TPJ in third-person perspective taking [85], [86], and the suggestion that TPJ plays a role in distinguishing one’s own body, actions, and feelings from those of perceived or imagined others [56], this might reflect increased efforts to distinguish one’s own emotions from those of the fictitious persons, since the latter are experienced more intensely. Although the lateralization of the TPJ cluster to the left hemisphere is incongruent with the fact that most of these accounts have focused on the right TPJ, recent suggestions indicate that the left TPJ is also involved in processing perspective differences [88]. Besides, at a more lenient intensity threshold (p<0.005) we also found a cluster in right TPJ. That said, this explanation is speculative and therefore we consider the interpretation of this result an open question.

### Interpretational Issues

Our findings confirm our central hypothesis that the perceived humanness of others is a crucial factor in moral decision making. In contrast to most previous perspective taking approaches (e.g. [55], [89]) the perspective-taking task with which we induced “perceived humanness” was implicit rather than explicitly instructed. Besides, the difference between the Humanized and Neutral priming trials was only subtle. As a consequence, participants seemed unaware of the manipulation. In this way we tried to minimize explicit reflections on the manipulation, which might have hindered spontaneous decision making. Furthermore, inducing our humanization manipulation prior to rather than during the decision period enabled us to keep the core characteristics of the dilemmas unchanged, which ensured that the modeled decision periods were comparable in all other aspects than the humanness of the primed person.

Although the humanization manipulation led to slightly but significantly more non-utilitarian responses, our imaging effects are independent of whether a utilitarian or non-utilitarian decision was made. There were not enough trials per cell (i.e. not every participant had made a non-utilitarian decision in both conditions) to directly test for interaction effects, but the imaging results of contrasting non-utilitarian versus utilitarian decisions did not overlap with those of the humanized versus neutral dilemma contrast (data not shown). Instead, contrasting non-utilitarian versus utilitarian decisions bilaterally activated the postcentral gyrus (S1), possibly since it involved a contrast between middle finger and index finger responses. Secondly, including the subject-specific differences between the number of utilitarian decisions in neutral and humanized dilemmas as a covariate in a multiple regression analysis contrasting humanized with neutral dilemmas did not alter the pattern of results. This indicates that the humanization effects cannot be explained by confounding effects of making a non-utilitarian decision and, for instance, the associated imagination of its aversive consequences. More generally, it suggests that our manipulation mainly acted on the decision *process.* When this decision process involved a humanized person, it was apparently more distressing, raised more negative affect and, possibly, also evoked attempts to override tendencies to decide non-utilitarian - irrespective of whether the final outcome of this process was an utilitarian choice or not.

Perspective taking has previously been shown to lead to more helping behavior, by inducing partiality (e.g. [90]). Our findings might shed more light on the mechanism underlying this effect, suggesting that the *result* of perspective taking, i.e., increased perceived humanness, might be the driving force behind its effects on prosocial behavior. Humanization, then, could affect prosocial behavior either by increasing empathic concern for the other, in turn increasing altruistic motivation [90], or by increasing the “perceived oneness” with the victims, and subsequent attempts to alleviate one’s own feelings of personal distress [91], [92]. Whether the effects of humanization on moral decision making are driven by increased empathic concern or increased personal distress could not be fully discerned by our findings and remains a question for future research.

The concept of humanization does not imply that a given person is explicitly considered to be “more human” than others – it only means that this person is more vividly perceived as a human being “like me” - with thoughts, feelings, and the ability to suffer. This altered, more pronounced perception of humanness would affect moral decision making since it affects the valuation of the imagined consequences of harmful decisions. That said, other factors - such as the extent to which the potential victim is able to take care of himself, or the extent to which others benefit from his being sacrificed, might counteract this effect. The present account also predicts that *decreasing* the extent to which others are perceived as having mental states that one can relate to, might lead to opposite effects on prosocial behaviour. Future work will be needed to confirm this.

### Conclusions

In the present study we have shown using a clear-cut experimental manipulation that increasing the extent to which others are perceived as human beings affects moral decisions about them. Our findings show that “human-like” persons are sacrificed less often than more neutrally perceived persons in moral dilemmas, and suggest that thinking about harming them is more distressing, leads to more empathic responses, evokes more emotional conflict, and hence more emotion regulation efforts and behavioral control. Thus, we suggest that the extent to which we perceive others as human is a driving force for - or against - prosocial behavior. Furthermore, humanization and its emotional and behavioral consequences might be a key variable in helping to resolve the longstanding riddle about non-utilitarian response tendencies raised by moral psychology.

## Supporting Information

Figure S1 **Example of a HUMANIZED priming trial.**
(PDF)

Figure S2 **Example of a NEUTRAL priming trial.**
(PDF)

Figure S3 **Example of a dilemma trial.**
(PDF)

## References

[1] Trivers RL (1971) The Evolution of Reciprocal Altruism. The Quarterly Review of Biology 46: 35–57.

[2] Wrangham RW, Peterson D (1996) Demonic males : apes and the origins of human violence; Peterson D, editor. Boston: Houghton Mifflin.

[3] Harcourt A, de Waal F (1992) Coalitions and alliances in humans and other animals. New York: Oxford University Press.

[4] Frith CD, Frith U (2006) The neural basis of mentalizing. Neuron 50: 531–534. 16701204 10.1016/j.neuron.2006.05.001

[5] Waytz A, Morewedge CK, Epley N, Monteleone G, Gao JH, et al (2010) Making sense by making sentient: effectance motivation increases anthropomorphism. J Pers Soc Psychol 99: 410–435. 20649365 10.1037/a0020240

[6] Haslam N (2006) Dehumanization: an integrative review. Pers Soc Psychol Rev 10: 252–264. 16859440 10.1207/s15327957pspr1003_4

[7] Leyens J-P, Paladino PM, Rodriguez-Torres R, Vaes J, Demoulin S, et al (2000) The Emotional Side of Prejudice: The Attribution of Secondary Emotions to Ingroups and Outgroups. Personality and Social Psychology Review 4: 186–197.

[8] Jahoda G (1999) Images of savages: ancient roots of modern prejudice in Western culture. London: Routledge.

[9] Allport G (1954) The nature of prejudice. Reading, MA: Addison-Wesley.

[10] Singer T, Lamm C (2009) The social neuroscience of empathy. Annals of the New York Academy of Sciences 1156: 81–96. 19338504 10.1111/j.1749-6632.2009.04418.x

[11] Decety J, Sommerville JA (2003) Shared representations between self and other: a social cognitive neuroscience view. Trends in cognitive sciences 7: 527–533. 14643368 10.1016/j.tics.2003.10.004

[12] Ciaramelli E, Muccioli M, Ladavas E, di Pellegrino G (2007) Selective deficit in personal moral judgment following damage to ventromedial prefrontal cortex. Soc Cogn Affect Neurosci 2: 84–92. 18985127 10.1093/scan/nsm001 PMC2555449

[13] Foot P (1967) The Problem of Abortion and the Doctrine of the Double Effect. Oxford Review 5: 5–15.

[14] Thomson JJ (1976) Killing, Letting Die, and the Trolley Problem. The Monist 59: 204–217. 11662247 10.5840/monist197659224

[15] Cushman F, Young L, Hauser M (2006) The role of conscious reasoning and intuition in moral judgment: testing three principles of harm. Psychological science : a journal of the American Psychological Society/APS 17: 1082–1089. 10.1111/j.1467-9280.2006.01834.x 17201791

[16] Greene JD, Cushman FA, Stewart LE, Lowenberg K, Nystrom LE, et al (2009) Pushing moral buttons: the interaction between personal force and intention in moral judgment. Cognition 111: 364–371. 19375075 10.1016/j.cognition.2009.02.001

[17] Moore AB, Clark BA, Kane MJ (2008) Who shalt not kill? Individual differences in working memory capacity, executive control, and moral judgment. Psychological science : a journal of the American Psychological Society/APS 19: 549–557. 10.1111/j.1467-9280.2008.02122.x 18578844

[18] Greene JD, Sommerville RB, Nystrom LE, Darley JM, Cohen JD (2001) An fMRI investigation of emotional engagement in moral judgment. Science 293: 2105–2108. 11557895 10.1126/science.1062872

[19] Greene JD, Nystrom LE, Engell AD, Darley JM, Cohen JD (2004) The neural bases of cognitive conflict and control in moral judgment. Neuron 44: 389–400. 15473975 10.1016/j.neuron.2004.09.027

[20] Borg J, Hynes C, Van Horn J, Grafton S, Armstrong W (2006) Consequences, Action, and Intention as Factors in Moral Judgments: An fMRI Investigation. Journal of cognitive neuroscience 18: 803–817. 16768379 10.1162/jocn.2006.18.5.803

[21] Cikara M, Farnsworth RA, Harris LT, Fiske ST (2010) On the wrong side of the trolley track: Neural correlates of relative social valuation. Social cognitive and affective neuroscience 5: 404–413. 20150342 10.1093/scan/nsq011 PMC2999760

[22] Harenski CL, Antonenko O, Shane MS, Kiehl KA (2008) Gender differences in neural mechanisms underlying moral sensitivity. Social cognitive and affective neuroscience 3: 313–321. 19015084 10.1093/scan/nsn026 PMC2607058

[23] Harenski CL, Hamann S (2006) Neural correlates of regulating negative emotions related to moral violations. NeuroImage 30: 313–324. 16249098 10.1016/j.neuroimage.2005.09.034

[24] Heekeren HR, Wartenburger I, Schmidt H, Schwintowski H-P, Villringer A (2003) An fMRI study of simple ethical decision-making. Neuroreport 14: 1215–1219. 12824762 10.1097/00001756-200307010-00005

[25] Mercadillo RE, Díaz JL, Pasaye EH, Barrios FA (2011) Perception of suffering and compassion experience: Brain gender disparities. Brain and Cognition 76: 5–14. 21492980 10.1016/j.bandc.2011.03.019

[26] Moll J, de Oliveira-Souza R, Bramati IE, Grafman J (2002) Functional Networks in Emotional Moral and Nonmoral Social Judgments. NeuroImage 16: 696–703. 12169253 10.1006/nimg.2002.1118

[27] Moll J, de Oliveira-Souza R, Eslinger PJ, Bramati IE, Mourão-Miranda Jn, et al (2002) The Neural Correlates of Moral Sensitivity: A Functional Magnetic Resonance Imaging Investigation of Basic and Moral Emotions. The Journal of Neuroscience 22: 2730–2736. 11923438 10.1523/JNEUROSCI.22-07-02730.2002 PMC6758288

[28] Moll J, Eslinger PJ, Oliveira-Souza Rd (2001) Frontopolar and anterior temporal cortex activation in a moral judgment task: preliminary functional MRI results in normal subjects. Arquivos de neuro-psiquiatria 59: 657–664. 11593260 10.1590/s0004-282x2001000500001

[29] Robertson D, Snarey J, Ousley O, Harenski K, Bowman FD, et al. (2006) The neural processing of moral sensitivity to issues of justice and care. Neuropsychologia. 10.1016/j.neuropsychologia.2006.08.014 17174987

[30] Shenhav A, Greene JD (2010) Moral Judgments Recruit Domain-General Valuation Mechanisms to Integrate Representations of Probability and Magnitude. Neuron 67: 667–677. 20797542 10.1016/j.neuron.2010.07.020

[31] Young L, Cushman F, Hauser M, Saxe R (2007) The neural basis of the interaction between theory of mind and moral judgment. Proceedings of the National Academy of Sciences 104: 8235–8240. 10.1073/pnas.0701408104 PMC1895935 17485679

[32] Young L, Saxe R (2009) An fMRI Investigation of Spontaneous Mental State Inference for Moral Judgment. Journal of cognitive neuroscience 21: 1396–1405. 18823250 10.1162/jocn.2009.21137

[33] Klein C (2011) The Dual Track Theory of Moral Decision-Making: a Critique of the Neuroimaging Evidence. Neuroethics 4: 143–162.

[34] Ugazio G, Lamm C, Singer T (2012) The role of emotions for moral judgments depends on the type of emotion and moral scenario. Emotion 12: 579–590. 21859189 10.1037/a0024611

[35] Harris LT, Fiske ST (2006) Dehumanizing the lowest of the low: neuroimaging responses to extreme out-groups. Psychological science : a journal of the American Psychological Society/APS 17: 847–853. 10.1111/j.1467-9280.2006.01793.x 17100784

[36] McGuire J, Langdon R, Coltheart M, Mackenzie C (2009) A reanalysis of the personal/impersonal distinction in moral psychology research. Journal of Experimental Social Psychology 45: 577–580.

[37] Greene JD (2009) Dual-process morality and the personal/impersonal distinction: A reply to McGuire, Langdon, Coltheart, and Mackenzie. Journal of Experimental Social Psychology 45: 581–584.

[38] Bechara A (2005) Decision making, impulse control and loss of willpower to resist drugs: a neurocognitive perspective. Nat Neurosci 8: 1458–1463. 16251988 10.1038/nn1584

[39] Koenigs M, Young L, Adolphs R, Tranel D, Cushman F, et al (2007) Damage to the prefrontal cortex increases utilitarian moral judgements. Nature 446: 908–911. 17377536 10.1038/nature05631 PMC2244801

[40] Singer T, Critchley HD, Preuschoff K (2009) A common role of insula in feelings, empathy and uncertainty. Trends in cognitive sciences 13: 334–340. 19643659 10.1016/j.tics.2009.05.001

[41] Lamm C, Singer T (2010) The role of anterior insular cortex in social emotions. Brain Struct Funct 214: 579–591. 20428887 10.1007/s00429-010-0251-3

[42] Lamm C, Decety J, Singer T (2011) Meta-analytic evidence for common and distinct neural networks associated with directly experienced pain and empathy for pain. NeuroImage 54: 2492–2502. 20946964 10.1016/j.neuroimage.2010.10.014

[43] Shackman AJ, Salomons TV, Slagter HA, Fox AS, Winter JJ, et al (2011) The integration of negative affect, pain and cognitive control in the cingulate cortex. Nat Rev Neurosci 12: 154–167. 21331082 10.1038/nrn2994 PMC3044650

[44] Ochsner KN, Ray RD, Cooper JC, Robertson ER, Chopra S, et al (2004) For better or for worse: neural systems supporting the cognitive down- and up-regulation of negative emotion. NeuroImage 23: 483–499. 15488398 10.1016/j.neuroimage.2004.06.030

[45] Ochsner KN, Gross JJ (2005) The cognitive control of emotion. Trends Cogn Sci 9: 242–249. 15866151 10.1016/j.tics.2005.03.010

[46] Ridderinkhof KR, van den Wildenberg WP, Segalowitz SJ, Carter CS (2004) Neurocognitive mechanisms of cognitive control: the role of prefrontal cortex in action selection, response inhibition, performance monitoring, and reward-based learning. Brain Cogn 56: 129–140. 15518930 10.1016/j.bandc.2004.09.016

[47] Langner O, Dotsch R, Bijlstra G, Wigboldus D, Hawk S, et al (2010) Presentation and validation of the Radboud Faces Database. Cognition and Emotion 24: 1377–1388.

[48] Sladky R, Friston KJ, Trostl J, Cunnington R, Moser E, et al (2011) Slice-timing effects and their correction in functional MRI. NeuroImage 58: 588–594. 21757015 10.1016/j.neuroimage.2011.06.078 PMC3167249

[49] Friston KJ, Holmes AP, Worsley KJ, Poline JP, Frith CD, et al (1994) Statistical parametric maps in functional imaging: A general linear approach. Hum Brain Mapp 2: 189–210.

[50] Penny WD, Holmes A (2004) Random-effects analysis. In: Penny W, Holmes A, Friston KJ, editors. Human Brain Function. San Diego: Elsevier. 843–850.

[51] Friston KJ, Holmes A, Poline JB, Price CJ, Frith CD (1996) Detecting Activations in PET and fMRI: Levels of Inference and Power. NeuroImage 4: 223–235. 9345513 10.1006/nimg.1996.0074

[52] Eickhoff SB, Stephan KE, Mohlberg H, Grefkes C, Fink GR, et al (2005) A new SPM toolbox for combining probabilistic cytoarchitectonic maps and functional imaging data. NeuroImage 25: 1325–1335. 15850749 10.1016/j.neuroimage.2004.12.034

[53] Friston KJ, Buechel C, Fink GR, Morris J, Rolls E, et al (1997) Psychophysiological and Modulatory Interactions in Neuroimaging. NeuroImage 6: 218–229. 9344826 10.1006/nimg.1997.0291

[54] Batson CD, Fultz J, Schoenrade PA (1987) Distress and empathy: two qualitatively distinct vicarious emotions with different motivational consequences. J Pers 55: 19–39. 3572705 10.1111/j.1467-6494.1987.tb00426.x

[55] Lamm C, Batson CD, Decety J (2007) The neural substrate of human empathy: effects of perspective-taking and cognitive appraisal. Journal of cognitive neuroscience 19: 42–58. 17214562 10.1162/jocn.2007.19.1.42

[56] Decety J, Lamm C (2007) The Role of the Right Temporoparietal Junction in Social Interaction: How Low-Level Computational Processes Contribute to Meta-Cognition. The Neuroscientist 13: 580–593. 17911216 10.1177/1073858407304654

[57] Vogt BA (2005) Pain and emotion interactions in subregions of the cingulate gyrus. Nat Rev Neurosci 6: 533–544. 15995724 10.1038/nrn1704 PMC2659949

[58] Picard N, Strick PL (1996) Motor areas of the medial wall: a review of their location and functional activation. Cerebral cortex 6: 342–353. 8670662 10.1093/cercor/6.3.342

[59] van Overwalle F, Baetens K (2009) Understanding others’ actions and goals by mirror and mentalizing systems: A meta-analysis. NeuroImage 48: 564–584. 19524046 10.1016/j.neuroimage.2009.06.009

[60] Gallagher HL, Frith CD (2003) Functional imaging of `theory of mind’. Trends in cognitive sciences 7: 77–83. 12584026 10.1016/s1364-6613(02)00025-6

[61] Gallagher HL, Happé F, Brunswick N, Fletcher PC, Frith U, et al (2000) Reading the mind in cartoons and stories: an fMRI study of `theory of mind’ in verbal and nonverbal tasks. Neuropsychologia 38: 11–21. 10617288 10.1016/s0028-3932(99)00053-6

[62] Siegal M, Varley R (2002) Neural systems involved in ‘theory of mind’. Nat Rev Neurosci 3: 463–471. 12042881 10.1038/nrn844

[63] Amodio DM, Frith CD (2006) Meeting of minds: the medial frontal cortex and social cognition. Nat Rev Neurosci 7: 268–277. 16552413 10.1038/nrn1884

[64] Saxe R, Carey S, Kanwisher N (2004) Understanding Other Minds: Linking Developmental Psychology and Functional Neuroimaging. Annual Review of Psychology 55: 87–124. 10.1146/annurev.psych.55.090902.142044 14744211

[65] Batson CD, Early S, Salvarani G (1997) Perspective Taking: Imagining How Another Feels Versus Imaging How You Would Feel. Personality and Social Psychology Bulletin 23: 751–758.

[66] Greene J, Haidt J (2002) How (and where) does moral judgment work? Trends in cognitive sciences 6: 517–523. 12475712 10.1016/s1364-6613(02)02011-9

[67] Anderson S, Bechara A, Damasio H, Tranel D, Damasio A (1999) Impairment of social and moral behavior related to early damage in human prefrontal cortex. Nature neuroscience 2: 1032–1037. 10526345 10.1038/14833

[68] Koenigs M, Tranel D (2007) Irrational economic decision-making after ventromedial prefrontal damage: evidence from the Ultimatum Game. J Neurosci 27: 951–956. 17251437 10.1523/JNEUROSCI.4606-06.2007 PMC2490711

[69] Kringelbach ML, Rolls ET (2004) The functional neuroanatomy of the human orbitofrontal cortex: evidence from neuroimaging and neuropsychology. Progress in Neurobiology 72: 341–372. 15157726 10.1016/j.pneurobio.2004.03.006

[70] Raine A, Yang Y (2006) Neural foundations to moral reasoning and antisocial behavior. Social cognitive and affective neuroscience 1: 203–213. 18985107 10.1093/scan/nsl033 PMC2555414

[71] Chang LJ, Sanfey AG (2008) Emotion, decision-making and the brain. Adv Health Econ Health Serv Res 20: 31–53. 19552303

[72] Damasio AR, Grabowski TJ, Bechara A, Damasio H, Ponto LL, et al (2000) Subcortical and cortical brain activity during the feeling of self-generated emotions. Nat Neurosci 3: 1049–1056. 11017179 10.1038/79871

[73] Wallis JD (2007) Orbitofrontal cortex and its contribution to decision-making. Annu Rev Neurosci 30: 31–56. 17417936 10.1146/annurev.neuro.30.051606.094334

[74] Diekhof EK, Geier K, Falkai P, Gruber O (2011) Fear is only as deep as the mind allows: A coordinate-based meta-analysis of neuroimaging studies on the regulation of negative affect. NeuroImage 58: 275–285. 21669291 10.1016/j.neuroimage.2011.05.073

[75] Etkin A, Egner T, Kalisch R (2011) Emotional processing in anterior cingulate and medial prefrontal cortex. Trends in cognitive sciences 15: 85–93. 21167765 10.1016/j.tics.2010.11.004 PMC3035157

[76] Craig A (2010) The sentient self. Brain Structure and Function 214: 563–577. 20512381 10.1007/s00429-010-0248-y

[77] Singer T, Seymour B, O’Doherty J, Kaube H, Dolan RJ, et al (2004) Empathy for pain involves the affective but not sensory components of pain. Science (New York, NY) 303: 1157–1162. 10.1126/science.1093535 14976305

[78] Valentini E (2010) The Role of Anterior Insula and Anterior Cingulate in Empathy for Pain. Journal of neurophysiology 104: 584–586. 20554847 10.1152/jn.00487.2010

[79] Mesulam MM, Mufson EJ (1982) Insula of the old world monkey. III: Efferent cortical output and comments on function. J Comp Neurol 212: 38–52. 7174907 10.1002/cne.902120104

[80] Cauda F, D’Agata F, Sacco K, Duca S, Geminiani G, et al (2011) Functional connectivity of the insula in the resting brain. NeuroImage 55: 8–23. 21111053 10.1016/j.neuroimage.2010.11.049

[81] Fletcher PC, Frith CD, Baker SC, Shallice T, Frackowiak RS, et al (1995) The mind’s eye–precuneus activation in memory-related imagery. NeuroImage 2: 195–200. 9343602 10.1006/nimg.1995.1025

[82] Vogt BA, Laureys S (2005) Posterior cingulate, precuneal and retrosplenial cortices: cytology and components of the neural network correlates of consciousness. 205–217 p. 10.1016/S0079-6123(05)50015-3 PMC2679949 16186025

[83] Cheetham M, Pedroni AF, Antley A, Slater M, Jancke L (2009) Virtual milgram: empathic concern or personal distress? Evidence from functional MRI and dispositional measures. Front Hum Neurosci 3: 29. 19876407 10.3389/neuro.09.029.2009 PMC2769551

[84] Farrow TF, Zheng Y, Wilkinson ID, Spence SA, Deakin JF, et al (2001) Investigating the functional anatomy of empathy and forgiveness. Neuroreport 12: 2433–2438. 11496124 10.1097/00001756-200108080-00029

[85] Jackson PL, Brunet E, Meltzoff AN, Decety J (2006) Empathy examined through the neural mechanisms involved in imagining how I feel versus how you feel pain. Neuropsychologia 44: 752–761. 16140345 10.1016/j.neuropsychologia.2005.07.015

[86] Decety J (2005) Perspective taking as the royal avenue to empathy. In: Malle BF, Hodges SD, editors. Other Minds: How Humans Bridge the Divide between Self and Others. New York: Guilford Publishers. 135–149.

[87] Wager TD, Feldman Barrett L (2004) From affect to control: Functional specialization of the insula in motivation and regulation.: Published online at PsycExtra: http://www.columbia.edu/cu/psychology/tor/. Accessed September 26, 2012

[88] Aichhorn M, Perner J, Weiss B, Kronbichler M, Staffen W, et al (2009) Temporo-parietal junction activity in theory-of-mind tasks: falseness, beliefs, or attention. J Cogn Neurosci 21: 1179–1192. 18702587 10.1162/jocn.2009.21082

[89] Lamm C, Decety J (2008) Is the extrastriate body area (EBA) sensitive to the perception of pain in others? Cereb Cortex 18: 2369–2373. 18270173 10.1093/cercor/bhn006

[90] Batson CD, Klein TR, Highberger L, Shaw LL (1995) Immorality From Empathy-Induced Altruism: When Compassion and Justice Conflict. Journal of personality and social psychology 68: 1042–1054.

[91] Maner JK, Luce CL, Neuberg SL, Cialdini RB, Brown S, et al (2002) The Effects of Perspective Taking on Motivations for Helping: Still No Evidence for Altruism. Personality and Social Psychology Bulletin 28: 1601–1610.

[92] Cialdini RB, Schaller M, Houlihan D, Arps K, Fultz J, et al (1987) Empathy-based helping: is it selflessly or selfishly motivated? J Pers Soc Psychol 52: 749–758. 3572736 10.1037//0022-3514.52.4.749

