# Supplementary material for: The Human Factor: Behavioral and Neural Correlates of Humanized Perception in Moral Decision Making
Source: PLoS One. 2012 Oct 17;7(10):e47698. doi: 10.1371/journal.pone.0047698 (PMC3474750; doi:10.1371/journal.pone.0047698)
Supplement: Figure S1 — Example of a HUMANIZED priming trial. (PDF) [file pone.0047698.s001.pdf]

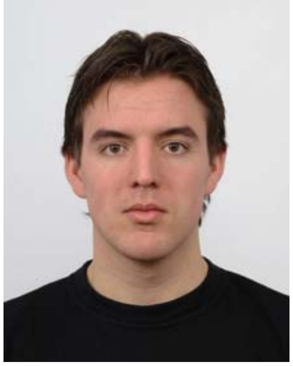

**Person A**

***A*** is a 28-year-old man who likes eating in good restaurants. Once again he has ordered shrimps – he felt that this choice fitted nicely to the restaurant and to his own taste. However, when a deliciously smelling lasagna dish is served at the table next to his, he suddenly doesn't feel like eating his shrimps anymore. Before he calls the waiter to change his order, he hesitates for a moment, pondering about whether it wouldn't be embarrassing to switch to a simple lasagne dish. But as soon as the lasagna is served, he is happy with his decision. He starts eating so hastily, that he burns his tongue. But that doesn't bother him – the smell of melted cheese and fresh oregano mean a lot more to him than the glassy shrimps.

Why, do you think, has ***A*** ordered shrimps in the first place?

1. Because he has eaten shrimps very often and couldn't foresee that he would suddenly prefer a different plate.
2. Because he felt it was embarrassing to order a more plain meal in such an expensive restaurant.

What, do you think, will ***A*** do after quickly eating his lasagna?

1. He will order another lasagne and will eat this one more slowly, because he finds the portion rather small.
2. He will order ice cream, to soothe his burnt tongue.
