# Supplementary material for: The Human Factor: Behavioral and Neural Correlates of Humanized Perception in Moral Decision Making
Source: PLoS One. 2012 Oct 17;7(10):e47698. doi: 10.1371/journal.pone.0047698 (PMC3474750; doi:10.1371/journal.pone.0047698)
Supplement: Figure S2 — Example of a NEUTRAL priming trial. (PDF) [file pone.0047698.s002.pdf]

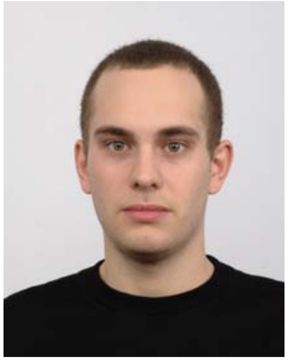

Person B

B is 27-year old man, who often goes cycling on the weekend. He mostly makes long trips with his bike, which is also suited for rocky undergrounds, due to its thick tyres with deep profile. He mostly does cross-country biking and then passes through a hilly area. For that reason, his bike has 24 gears. When it rains, he often makes a smaller tour and only rides on roads. Then it is better to pump up the tyres a bit harder. He always locks the bike if he leaves it somewhere, because it was very expensive and bikes get stolen quite often. One year ago, his bike was stolen. Fortunately it was insured and he could easily replace it. His new bike is almost identical, only the colour is now blue instead of green.

Question: Why, do you think, does ***B*** use a higher pressure for his tyres when he rides on roads?
1. Because then the bike has less roll resistance.
2. Because then the tyres don't need to absorb the bumps in the terrain.

How many gears do you think do most city bikes have?
1. Meanwhile, most city bikes have 21 gears or more.
2. Most city bikes clearly have less than 21 gears.
