# Supplementary material for: The Human Factor: Behavioral and Neural Correlates of Humanized Perception in Moral Decision Making
Source: PLoS One. 2012 Oct 17;7(10):e47698. doi: 10.1371/journal.pone.0047698 (PMC3474750; doi:10.1371/journal.pone.0047698)
Supplement: Figure S3 — Example of a dilemma trial. (PDF) [file pone.0047698.s003.pdf]

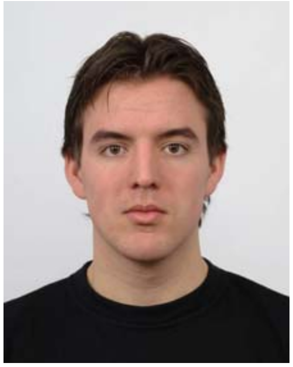

Person A

Flap bridge

You control a flap bridge and have just opened the bridge. A boat, in which one of the boatmen, ***A***, is sitting on the mast beam, is passing under the bridge. Suddenly you see that a car, whose driver apparently overlooked a stop signal, is already on the tilted bridge. If you do not act, the car will soon be falling off the bridge which is tilting further and further. This would result in the death of or at least severe injuries for the five passengers.

Although you cannot stop the bridge from opening, you can change the bridge's direction by turning a lever, so that the bridge closes again. This would prevent the car from falling off the bridge, and its passengers would be saved. However ***A***, who is sitting on the mast beam and cannot leave the boat in time, would be crushed by the bridge and would be at least severely hurt. If you do not act, the car with its five passengers will fall off the bridge, but the boatman, ***A***, will be spared.

Will you turn the lever to change the direction of the bridge?

Yes   No
